# Direct and indirect transmission of four *Salmonella enterica *serotypes in pigs

**DOI:** 10.1186/1751-0147-52-30

**Published:** 2010-05-10

**Authors:** Julia Österberg, Susanna Sternberg Lewerin, Per Wallgren

**Affiliations:** 1 National Veterinary Institute, SVA, SE-751 89 Uppsala, Sweden; 2 Department of Clinical Sciences, Swedish University of Agricultural Sciences, SE-750 07 Uppsala, Sweden

## Abstract

**Background:**

Feed-borne spread of *Salmonella *spp. to pigs has been documented several times in recent years in Sweden. Experiences from the field suggest that feed-associated serotypes might be less transmittable and subsequently easier to eradicate from pig herds than other serotypes more commonly associated to pigs. Four *Salmonella *serotypes were selected for experimental studies in pigs in order to study transmissibility and compare possible differences between feed-assoociated (*S *Cubana and *S *Yoruba) and pig-associated serotypes (*S *Derby and *S *Typhimurium).

**Methods:**

Direct contact transmission was studied in four groups of pigs formed by six 10-week-old salmonella negative pigs commingled with two fatteners excreting one of the four salmonella serotypes. Indirect transmission was studied by putting six 10-week-old salmonella negative pigs in each of four salmonella contaminated rooms. Each room had previously housed a group of pigs, excreting one of the four selected serotypes.

All pigs were monitored for two weeks with respect to the faecal excretion of salmonella and the presence of serum antibodies. At the end of the trial, eight samples from inner tissues and organs were collected from each pig at necropsy.

**Results:**

In the four direct transmission groups, one pig shed *Salmonella *(Cubana) at one occasion. At necropsy, *S *Typhimurium was isolated from one pig.

In the indirect transmission groups, two pigs in the Yoruba room and one pig in each of the other rooms were excreting detectable levels of *Salmonella *once during the study period of two weeks. At necropsy, *S *Derby was isolated from one of six pigs in the Derby room and *S *Typhimurium was isolated from four of the six pigs in the Typhimurium room.

No significant serological response could be detected in any of the 48 pigs.

**Conclusions:**

These results show that all four selected serotypes were able to be transmitted in at least one of these field-like trials, but the transmission rate was low in all groups and no obvious differences between feed-associated and pig-associated serotypes in the transmission to naïve pigs and their subsequent faecal shedding were revealed. However, the post mortem results indicated a higher detection of *S *Typhimurium in the ileocecal lymph nodes of pigs introduced into a contaminated environment in comparison with the other three serotypes.

## Background

All serotypes of *Salmonella *are considered a potential health hazard to humans [1,2]. The serotypes commonly detected in raw feed materials in Sweden often differ from the serotypes most often isolated from animals and humans. However, in recent years some of these "feed-associated" serotypes have been detected in pig herds [3-5]. Changes in the feed industry, with a higher proportion of imported raw feed materials with a higher prevalence of *Salmonella *spp. [6], might be an explanation for this detection of "new" serotypes in Swedish pig herds.

Why only few of the "feed-associated" serotypes seem to reach and/or spread within the animal population has not been sufficiently investigated, although consequent monitoring of imported raw feed materials for presence of *Salmonella*, with subsequent heat- and acid treatments whenever detected, certainly contribute to this. Most serotypes detected in raw feed materials or in feed plants have not been studied in animals under experimental conditions. The transmission of *Salmonella *in pigs seems to be serovar related [7]. Also, experiences from the field suggest that some serotypes are more transient in the pig than others. However, if this is a matter of virulence factors, dose-response characteristics or due to other epidemiological differences are not fully known.

According to the Swedish law, action must be taken to eliminate the infection or contamination with *Salmonella *spp. whenever the bacterium is detected in the food chain of animal products (Zonoosis Act, SFS 1999:658). This means that when *Salmonella *spp. is detected in a herd, the herd is put under restrictions prohibiting animals to leave the farm before all infected animals have been culled or tested negative in two feacal samples taken one month apart. Furthermore, all contaminated surfaces on the premises must be thoroughly cleaned and disinfected before the restrictions can be lifted. With increasing herd sizes in modern agriculture the costs for the control of *Salmonella *at herd level are increasing, emphasizing the need for more cost-efficient eradication measures.

In previous studies we have compared the two feed-associated serotypes *S *Yoruba and *S *Cubana with the two "classic" serotypes *S *Typhimurium and *S *Derby, as regards infectious dose, faecal excretion pattern, antibody response and distribution in internal organs and tissues [8,9] with the aim to elucidate possible differences between serotypes that might be of significance for *Salmonella *control strategies.

In the present study we continued the comparative examinations of these four selected serotypes by studying their ability to infect pigs through direct and indirect transmission, intending to resemble the conditions present in pig herds. The specific aim of these experiments was to investigate if the results obtained would support the idea of adapting herd level eradication strategies to different serotypes of *Salmonella*.

## Methods

### Animals and experimental facilities

Pigs (Yorkshire x Swedish Landrace) aged 10 weeks were bought from a Swedish nucleus herd, with a well documented high health status. The herd was regularly monitored for *Salmonella *spp. by bacteriological analysis of faecal samples. The herd was also declared free from atrophic rhinitis, mange, swine dysentery and PMWS by domestic control programs. Furthermore, Sweden is free from PRRS and Aujeszky's disease.

On two occasions, 24 pigs, originally derived from six litters, were transported to the research facilities at the National Veterinary Institute (NVI) in Uppsala and split into four groups on arrival, each group including one pig from each of the six litters.

Each group was housed in a pen in a separate room, as previously described in detail [8]. The rooms were separated also with respect to the ventilation and the manure systems. The pen in each room had a solid concrete floor with sawdust bedding covering half of the pen. The dung was cleared out twice daily. The pigs were fed a pelleted fattening feed (Origo 522 PK; Lantmännen, Svalöv, Sweden).

### Experimental design

The experiments were approved by the ethical committee for animal experiences in Uppsala, Sweden (licence C264/5).

*Direct contact transmission (DT) *was studied by mixing six pigs with two seeder pigs, aged 18 weeks, originally from the same herd as the younger pigs, in each of four cleaned and disinfected pens (in total 24 naïve pigs and eight seeder pigs). The two seeder pigs in each group had a well documented history of long-term feacal excretion of one of the four serotypes of *Salmonella*, as they had been part of another study and inoculated with 10^9 ^colony forming units (CFU) of the serotype eight weeks earlier [8,9].

*Indirect transmission (IT) *was studied in six pigs in each of four pens (in total 24 naïve pigs), where *Salmonella *infected pigs had previously been housed as part of another study. In that former study, six pigs in each pen had been inoculated with 10^9 ^CFU of one of the four serotypes studied and their faecal excretion of *Salmonella *had been monitored for eight weeks [8,9]. The inoculated pigs left the pens the same morning as the new, uninfected pigs were introduced to the same, scraped but not washed, pens.

In both the direct and the indirect transmission study, the pigs were monitored for two weeks, with respect to the excretion of *Salmonella *in faeces and the presence of antibodies in serum. Further sampling to demonstrate presence of *Salmonella *was carried out at necropsy at the end of the trial.

### Collection of samples

Individual faecal samples were collected on arrival to the experimental facilities at the NVI, prior to the commingling with the seeder pigs or the introduction to the contaminated pens, respectively (Day 0). Thereafter, faecal samples were collected daily for four days (Day 1, 2, 3 and 4) and four times during the second week (Day 7, 9, 11 and 14).

Blood samples were collected into plain tubes from all pigs on Day 0, 7 and 14. The serum was removed and stored at -20°C until analysed.

All pigs were euthanised and necropsied on Day 14. At necropsy, tissue samples were collected from each pig from the following eight sites; tonsil, liver, spleen, colonic tissue, caecum contents and three lymph nodes (the mandibular, ileocecal and colonic lymph nodes).

### Qualitative culture of *Salmonella *species

Qualitative analyses were initiated within a couple of hours after the collection of faecal samples, using modified semi-solid Rappaport Vassiliadis (MSRV) selective enrichment medium (Draft Amendment ISO 6579: 2002).

The organ samples were cut with a scalpel into small pieces, before they were pre-enriched in a 1:9 dilution of BPW (CM0509; Oxoid) and further analysed in the same way as the faecal samples. The confirmation of the serotypes was made by direct agglutination, as previously described [8].

### Detection of serum antibodies

Serum antibodies to *S *Typhimurium were detected with a commercial ELISA (Svanovir; Svanova Biotech). Antibodies to *S *Yoruba were analysed with an in-house ELISA [8].

Serum antibodies to the two serotypes *S *Cubana and *S *Derby were analysed with a commercial ELISA (Herdchek Swine Salmonella, IDEXX Laboratories) based on LPS-antigens from *Salmonella *serogroups B, C1 and D.

## Results

### Direct contact transmission (DT)

**Figure 1:**
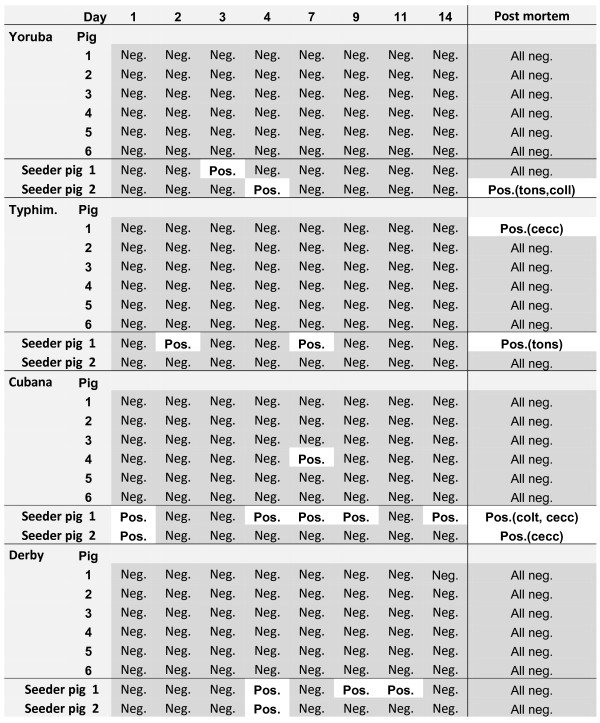
**The individual faecal shed of *Salmonella *in pigs following mixing of two seeder pigs with six naive pigs (DT-study)**. The two seeder pigs in each group were inoculated with 0.65 × 10^9 ^cfu of one of four different serovars of *Salmonella *eight weeks earlier. Demonstration of *Salmonella *is illustrated by white, whereas grey illustrates that *Salmonella *was not detected. In the right column the results from analysis of *Salmonella *in tissue samples taken post mortem are shown. Within brackets the tissue from which *Salmonella *was isolated are abbreviated as follows: cecum content = cec, colon lymph node = col, colon tissue = cot, tonsil = ton.

In all four DT groups, *Salmonella *was isolated from at least one of the two seeder pigs in at least one faecal sample collected during the study period of two weeks (Figure 1). Taken together, seven of the eight seeder pigs were *Salmonella *positive in 14 of the totally 64 samples collected. Except for one weaner shedding *S *Cubana on day 7, the younger pigs were all *Salmonella *negative in faeces during the entire period studied (Figure 1).

All pigs were seronegative to *Salmonella *prior to the study. In the DT trials with *S *Typhimurium, *S *Derby and *S *Cubana all pigs remained serologically negative.

The in-house ELISA constructed to detect antibodies against *S *Yoruba resulted in two pigs with an antibody level just above the cut-off at A_450 _= 0.60 (the highest reaction being 0.72).

The results from the culture of the post mortem samples are shown in Figure 1. Four of the eight seeder pigs had at least one post mortem sample from which the expected serotype of *Salmonella *was isolated. One weaner in the DT Typhimurium group was positive for *S *Typhimurium in the caecum content. All other samples from the 24 naive pigs in the DT study were *Salmonella *negative (n = 191) (Figure 1).

All the *Salmonella *positive cultures were confirmed by direct agglutination and in each sample the expected serotype was isolated.

### Indirect contact transmission (IT)

**Figure 2:**
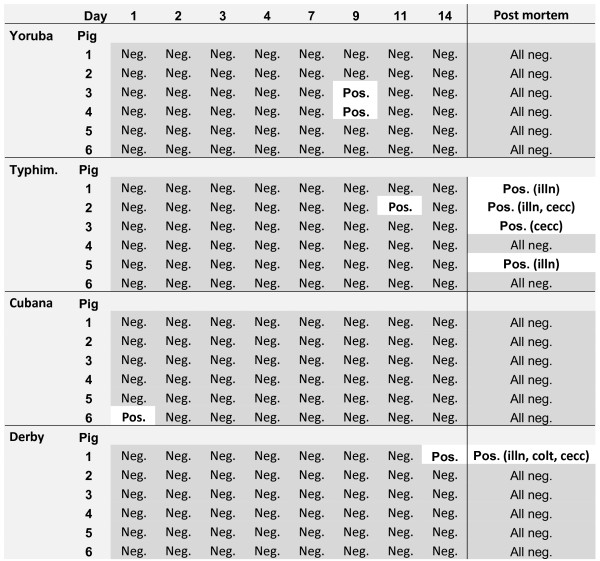
**The individual faecal shed of *Salmonella *in six pigs after their introduction into *Salmonella *contaminated pens (IT-study)**. The pens had harboured *Salmonella *shedding pigs, individually inoculated with 0.65 × 10^9 ^cfu of *Salmonella *eight weeks earlier. The inoculated pigs were removed and replaced with the naive, 10 weeks old pigs. Demonstration of *Salmonella *is illustrated by white, whereas grey illustrates that *Salmonella *was not detected. In the right column the results from analysis of *Salmonella *in tissue samples taken after euthanisation are shown. Within brackets the tissue from which *Salmonella *were isolated are abbreviated as follows: cecum content = cec, colon tissue = cot, ileocecal lymph node = iln.

Two pigs located in the pen contaminated with *S *Yoruba shed *S *Yoruba once during the experimental period of two weeks (Figure 2). In each of the other three groups (in pens contaminated with *S *Typhimurium, *S *Cubana and *S *Derby, respectively) *Salmonella *was detected in faeces from one pig on one occasion.

All pigs were seronegative to *Salmonella *when entering the contaminated facilities. All pigs that had been housed in pens contaminated with *S *Typhimurium, *S *Derby or *S *Cubana remained serologically negative throughout the study. The in-house ELISA resulted in two pigs in the Yoruba room with serological reactions just above the cut-off at A_450 _= 0.60 (the highest reaction being 0.82).

*S *Typhimurium was demonstrated in at least one post mortem sample from four of the six pigs in the *S *Typhimurium contaminated pen. Three of these four pigs had *S *Typhimurium isolated from the ileocecal lymph node. One pig in the *S *Derby contaminated pen was positive for *S *Derby in three of the eight samples collected at necropsy, while the other pigs in that group as well as all pigs from the *S *Cubana and *S *Yoruba contaminated rooms were all *Salmonella *negative in the post mortem samples (Figure 2).

All the *Salmonella *positive bacteriological samples were confirmed by direct agglutination and in each sample the expected serotype was isolated.

## Discussion

*Salmonella *appeared not to be readily transmitted to the naive pigs in these trials, neither from the seeder pigs, nor from the contaminated pens. Nevertheless, *Salmonella *was transmitted to at least one pig in all groups except in two of the direct contact groups (DT-trial; *S *Yoruba and *S *Derby). The division into a direct and an indirect transmission was made to mirror two different field situations, both expected to be of importance in an infected herd. The pathogen load appeared to be higher in the contaminated pens than from the intermittently shedding seeder pigs at a late stage of infection. However, the rate of transmission was low in both the direct and the indirect transmission situation and no differences between the four serotypes could be concluded with respect to the faecal shedding of *Salmonella*. These trials were performed in facilities with a high level of biosecurity, allowing only a limited number of pigs. This reduced the possibility to detect small differences between serotypes, but the design of the study was still regarded to be sufficient for its purpose.

Still, a difference between serotypes was indicated by the results obtained post-mortem as *S *Typhimurium was re-isolated from the majority of the pigs that had been housed in the contaminated environment. The most common site for the detection of *S *Typhimurium was the ileocecal lymph node. In concordance with our results, eight of nine pigs in another study were positive for *S *Typhimurium in the ileocolic lymph node three weeks after mixing with inoculated, *Salmonella*-shedding pigs, although most of the pigs were negative in rectal swabs collected during the trial period of three weeks [10]. Several other studies have also shown the affinity for the ileocecal/ileocolic lymph nodes of *S *Typhimurium [11-13]. Conversely, the feed-associated serotypes *S *Yoruba and *S *Cubana were not demonstrated in any lymphatic or other, non-intestinal, tissues, well in accordance with our previous results [8,9].

A number of studies have shown a rapid transmission of *Salmonella *spp. to pigs put in a contaminated environment [14-17]. These studies have mainly focused on the transmission of *Salmonella *occurring at transport and in lairage at abattoirs. The time frame of interest was then counted in hours, but the situation for the pigs was crowded and stressful which raises the risk for spread of the infection [18,19]. Our study aimed to resemble the situation at herd level to investigate the risk for transmission to naive pigs when exposed to contaminated pens or to infected pen mates. Thus, the density of pigs and the stress level were lower, which may have contributed to the low rate of *Salmonella *transmission despite the fairly long exposure period. Yet, it can not be ruled out that a longer study period than two weeks would have led to a more thorough transmission of the pathogen.

Above all, the contamination level in the environment is probably the most important factor affecting the spread of *Salmonella *in a pen. A difference in infection rate of pigs following exposure to pens contaminated with different levels of *S *Typhimurium has been reported [14]. Still, no attempt to quantify the contamination level of the pens was performed in the present study, due to an expected uneven distribution of *Salmonella *in the contaminated pens with a subsequent difficulty to define the true pathogen load. However, the shedding of *Salmonella *had been considerable in the pigs that had been housed in the pens in the previous trials [8,9]. This ought to have guaranteed a thorough contamination of the pens for the study of indirect transmission, especially since they were not washed before the introduction of naive pigs. Further, the water nipple in each pen was placed in the corner used as dunging area and the naive pigs drank from the floor until they had learnt to use the nipple properly. The one pig shedding *S *Cubana the second day in the pen was probably a result of such drinking.

In the DT-study, a majority of the seeder pigs showed signs of reduced shedding as eight weeks had passed since the inoculation of 10^9 ^CFU [8,9]. Also in this trial no attempts to estimate the challenge dose were performed. The qualitative analysis was regarded good enough, as our main interest was to measure if the numbers of bacteria were enough to reach above the threshold for detectable infection. Also, to quantify the concentration of *Salmonella *in the faeces of the seeder pigs was deemed not very useful as it is variable and the dose also would have been affected by the build-up of environmental contamination. As the direct transmission was studied in previously cleaned and disinfected facilities the pathogen load was probably low. However, one of the *S *Cubana seeder pigs had a history of constant faecal shedding in all the 23 faecal samples collected during the eight weeks prior to the commingling with the naive pigs [9] and continued to shed *S *Cubana in five of the eight faecal samples collected during the study period of two weeks. This was also the only group where faecal shedding of *Salmonella *was demonstrated in the weaners in the DT-study. Thus, it would appear that a pig shedding *Salmonella *in faeces, at a detectable but low level does not necessarily transmit the pathogen to its pen-mates, even in a solid floor environment.

Several studies show that factors such as poor pen cleaning, stress, concurrent disease, overcrowding and different feeding regimes are important for the infection dynamics in a herd [18-21]. In addition, the rate of transmission of *Salmonella *in a herd may be serotype-related. The pig-adapted serotype *S *Derby, for example, has managed to survive in a pig herd for more than a decade, in spite of several eradication attempts [22]. In contrast, only very few reoccurrences of *Salmonella *were demonstrated after the initial diagnosis of *S *Cubana in faeces in 31 farms, following a feed-borne spread of *S *Cubana in 2003 [4]. In the present study, the culture results from the necropsy of the IT-Typhimurium group (with four out of six pigs being positive for *S *Typhimurium) may have mirrored a higher pathogen load in this room, but more probably reflected that this serotype differs from the other three serotypes in a low-contaminated environment.

In conclusion, the low level of transmission of all four serotypes in both trials most likely reflected an overall low dose exposure and mirrored the dose-response relationship that has been shown in previous studies [8,9,23]. Therefore, these results high-lights the importance of hygienic measures rather than indicate a possibility for the adaptation of control strategies to different serotypes. A thorough cleaning and disinfection of units between batches of pigs can reduce the contamination level of the pen below the infection threshold, which may prevent the transmission of *Salmonella *spp. to the subsequent batch of pigs. This could be an important step to decrease the prevalence at slaughter, provided that the pigs are not infected/contaminated at a later stage, i.e. during transport, lairage or slaughter. Thus, hygienic measures in order to reduce the level of contamination in the environment in all stages of the animal-food chain still constitute the basis for the control of *Salmonella spp*.

## Conclusions

The present study showed a transmission of all four selected serotypes to the naive pigs in the contaminated environment and for two of the serotypes (*S *Cubana and *S *Typhimurium) in the direct contact situation. However, it did not indicate any obvious differences between them in their transmissibility, but rather showed a low transmission for all four serotypes studied. Neither did pigs that shed *Salmonella *necessarily transmit the pathogen to its pen-mates, nor did a *Salmonella*-contaminated environment inevitably lead to infected pigs.

These results high lights the importance of high hygienic standards in the environment of the pigs, in order to reduce the risk for high numbers of *Salmonella *to accumulate in the surroundings of the pigs. Nevertheless, in contrast to the other serotypes *S *Typhimurium was re-isolated from the ileocecal lymph nodes of three out of six pigs at necropsy, indicating a difference in the ability to infect a pig in a low-contaminated environment.

## Competing interests

The authors declare that they have no competing interests.

## Authors' contributions

JÖ was the co-ordinator involved in all parts of the study. JÖ also performed the collection of samples, participated in the laboratory work and was the main author of the manuscript. PW and SSL contributed to the design and planning and made substantial contributions in revising the manuscript critically. All authors have read and approved the final manuscript.
